# Precise and automated lung cancer cell classification using deep neural network with multiscale features and model distillation

**DOI:** 10.1038/s41598-024-61101-7

**Published:** 2024-05-07

**Authors:** Lan Tian, Jiabao Wu, Wanting Song, Qinghuai Hong, Di Liu, Fei Ye, Feng Gao, Yue Hu, Meijuan Wu, Yi Lan, Limin Chen

**Affiliations:** 1 Department of Pulmonary and Critical Care Medicine, Fujian Medical University Union Hospital, Fuzhou, 350001 Fujian China; 2 Department of Pathology, The First Affiliated Hospital of Fujian Medical University, Fuzhou, 350005 Fujian China; 3 Department of Oncology, Guang’anmen Hospital, China Academy of Chinese Medical Sciences, Beijing, 100053 China; 4 Department of Pulmonary and Critical Care Medicine, The Second Hospital of Sanming, Sanming, 366000 Fujian China; 5 Department of General Medicine, Nanping First Hospital Affiliated to Fujian Medical University, Nanping, 353000 Fujian China

**Keywords:** Lung cancer, Computational models

## Abstract

Lung diseases globally impose a significant pathological burden and mortality rate, particularly the differential diagnosis between adenocarcinoma, squamous cell carcinoma, and small cell lung carcinoma, which is paramount in determining optimal treatment strategies and improving clinical prognoses. Faced with the challenge of improving diagnostic precision and stability, this study has developed an innovative deep learning-based model. This model employs a Feature Pyramid Network (FPN) and Squeeze-and-Excitation (SE) modules combined with a Residual Network (ResNet18), to enhance the processing capabilities for complex images and conduct multi-scale analysis of each channel's importance in classifying lung cancer. Moreover, the performance of the model is further enhanced by employing knowledge distillation from larger teacher models to more compact student models. Subjected to rigorous five-fold cross-validation, our model outperforms existing models on all performance metrics, exhibiting exceptional diagnostic accuracy. Ablation studies on various model components have verified that each addition effectively improves model performance, achieving an average accuracy of 98.84% and a Matthews Correlation Coefficient (MCC) of 98.83%. Collectively, the results indicate that our model significantly improves the accuracy of disease diagnosis, providing physicians with more precise clinical decision-making support.

## Introduction

Lung cancer is a very aggressive and highly prevalent disease worldwide, with an estimated 2.2 million new cases and 1.8 million deaths in 2020^1^. Primary lung cancers are divided into two major types: small cell lung cancer and non-small cell lung cancer. Recent improvements in chemotherapy and radiation therapy^2^ have resulted in the latter being further classified into adenocarcinoma, squamous cell carcinoma, and large cell carcinoma^3^. Non-small cell lung cancer (NSCLC) is the predominant subtype, accounting for 85% of the cases^4^. Small cell lung cancer, an aggressive form with high mortality, meanwhile accounts for 15% of the cases^5^. The stage of the patient diagnosed with lung cancer is normally intermediate to advanced stage, the current medical level of clinical surgery has not very effective. Masaya Yotsukura et al.^6^ reviewed the pathologic findings of 524 patients with curative resection for adenocarcinoma in situ (AIS) and minimally invasive adenocarcinoma (MIA) who underwent resection for lung cancer. The study found that Estimated 10-year postoperative disease-specific survival rates were 100% and 100%, and overall survival rates were 95.3% and 97.8% for AIS and MIA cases. Therefore, if early-stage NSCLC patients can be accurately identified, this will help to slow down the progression of the disease and improve the quality of life of the individual.

Histopathologic confirmation remains the gold standard in clinical workflow for diagnosis^7^. Mukhopadhyay et al.^8^ compared the diagnostic performance of digital pathology (DP) and traditional microscopy-based methods in a study with specimens from 1992 patients, evaluated by 16 surgical pathologists from four institutions. The study found that DP was non-inferior to conventional microscopy for primary diagnosis in surgical pathology. Coudray et al.^9^ trained inception-v3 with a large number of image data from TCGA histopathological images. The appearance of this model can preliminarily predict gene mutation by only inputting H&E stained images, overcome the shortcomings of human naked eye recognition and summary of features, and has the advantages of low cost and high efficiency. The introduction of AI-based tools, with their power to unlock pathological diagnostic, prognostic, and predictive features, could assist pathologists, pulmonologists, and thoracic oncologists to guide patient management acting as a decision support system for pathologists^10^.

Deep Learning (DL) is one of the most critical factors for AI's success. It has a strong degree of automation. Ojansivu et al.^11^ investigated automated classification of breast cancer from histopathological images. Xu et al. developed a deep convolutional neural network that segments and classifies epithelial and stromal regions in histopathological images. Litjens et al. investigated the effect of DL for histopathological examination and verified that its performance was excellent in prostate cancer identification and breast cancer metastasis detection^12^. Compared with traditional algorithms, when the amount of data processed is too large, deep learning algorithms are slower than traditional algorithms, and a series of problems are prone to occur in practical applications. Therefore, we urgently need a more stable and processing power algorithm model. Knowledge distillation (KD) has proven to be a highly effective approach for enhancing model performance through a teacher-student training scheme^13,14^. KD trains a tiny student model to learn knowledge from a large pre-trained teacher model. This improves the performance of the network without significantly increasing the computational cost. In this paper, we also introduce a new architectural unit, which we term the Squeeze-and Excitation (SE) block, with the goal of improving the quality of representations without significantly increasing the computational cost. Many past studies^15–18^ have shown that feature maps in a feature pyramid can capture an object’s visual features at different scales. In this study, we developed an automated classification scheme for lung cancers in microscopic images and it was markedly improved with screening of the above procedures.

In the field of histopathology, distinguishing between adenocarcinoma and squamous cell carcinoma of the lung is often challenging, necessitating specialized training for pathologists. The development of an AI-based, precise, automatic, and rapid diagnostic model that can quickly and in bulk identify lung adenocarcinoma, squamous cell carcinoma, or benign lung tissue holds substantial clinical value. Such a model could assist physicians in making swift disease diagnoses, thereby benefiting subsequent treatment strategies. In our research, we introduce a deep learning network model tailored for lung cancer cell classification. Utilizing a teacher-student framework, our student baseline model adopts the Resnet18 network architecture. To enhance the capture of multi-scale features, we have incorporated a Feature Pyramid Network (FPN) for multi-scale feature extraction and added an SE module between Resnet18 and FPN. The SE module dynamically adjusts the channel weights of the feature maps through learned weights, augmenting the model's selective capture of information. The teacher model employs a pre-trained Resnet50 architecture. To validate the effectiveness of our model, we conducted ablation studies to rigorously assess the impact of each improvement measure. In addition to ablation experiments, comparative experiments were carried out against traditional image classification models capable of categorizing lung cancer cells [VIT, ResNet101, MobileNet, convolutional neural network (CNN)], aiming to demonstrate the superiority of our proposed tri-classification model for lung adenocarcinoma, squamous cell carcinoma, and benign lung tissue. The experiments indicate that our proposed model achieves optimal classification accuracy.

## Methods

### Data acquisition

**Figure 1:**
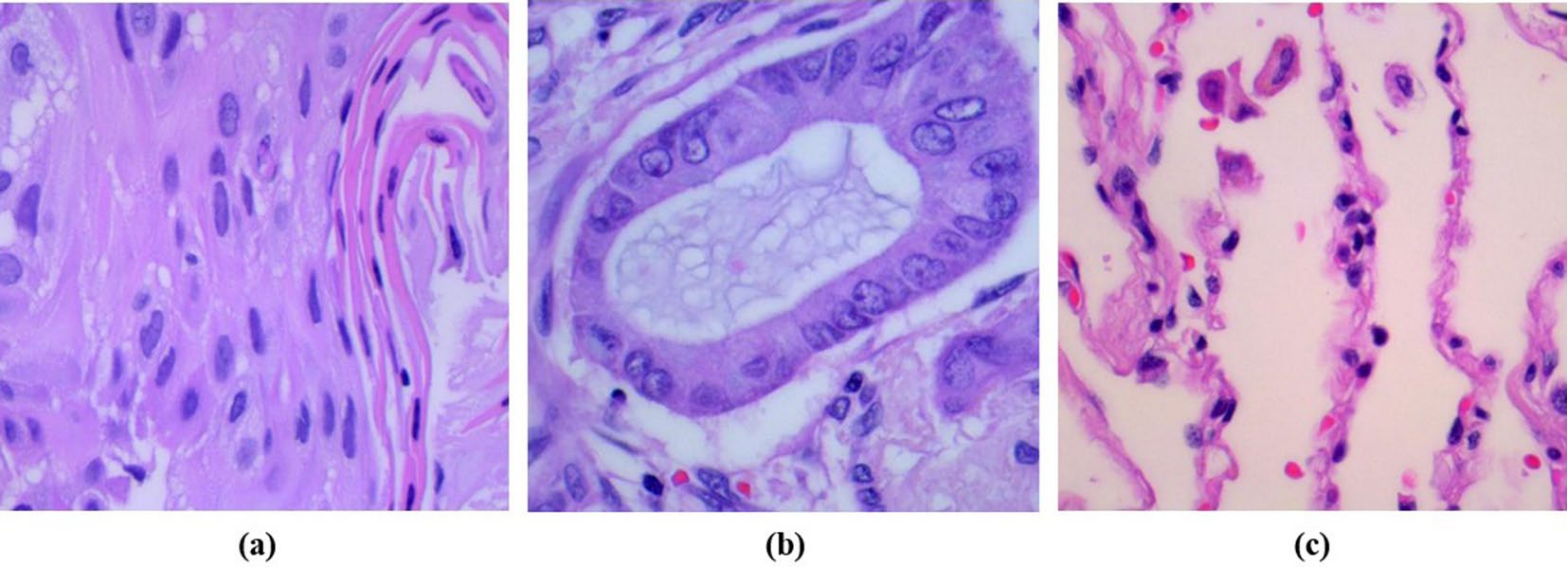
The dataset examples are shown below: image (**a**) representing lung adenocarcinoma, image (**b**) showing lung squamous cell carcinoma, and image (**c**) depicting normal cells.

The dataset used in this research is a publicly available collection from Kaggle^19^, containing 25,000 histopathological images divided into five categories. This set includes 250 images each of benign lung tissue, lung adenocarcinoma, and lung squamous cell carcinoma, along with 500 images related to colon tissue. Each image is uniformly sized at 768 × 768 pixels and is in JPEG format. Our study focuses on the classification and recognition of lung cancer, thus removing images of colon tissue. The dataset is exemplified in Fig. 1, with image (a) representing lung adenocarcinoma, image (b) showing lung squamous cell carcinoma, and image (c) depicting normal cells.

Lung adenocarcinoma, a leading subtype of non-small cell lung cancer, arises from the epithelial cells of the alveoli and is typically found on the lung's outer edges. Under the microscope, it presents diverse growth patterns, including acinar, papillary, lepidic, and solid formations, often with mucin secretion. Diagnosis commonly depends on specific immunohistochemical staining, like positive TTF-1 expression. Lung adenocarcinoma may also exhibit genetic markers such as mutations or rearrangements in EGFR, KRAS, ALK, and ROS1, valuable for targeted therapies. Lung squamous cell carcinoma typically develops in the central bronchi and has a strong link to smoking. Its cells display pronounced squamous characteristics, including keratinization (keratin pearls inside cells) and intercellular bridges. These tumors are usually hard and may have areas of central necrosis. Positive p63 or p40 immunohistochemical staining assists in distinguishing it from other non-small cell lung cancer subtypes. Squamous cell carcinoma often has fewer genetic changes linked to non-smoking but may include molecular variations like PIK3CA, SOX2.

### Data preprocessing

In this study, to enhance the generalization capability of our model in classifying lung cancer images, we implemented a variety of data augmentation techniques, thereby improving its robustness in practical applications. For the training dataset, we executed a series of randomized image processing steps. These included random cropping and resizing images to a uniform size of 224 × 224 pixels, random horizontal flipping, and random rotations of up to 10 degrees, to simulate transformations encountered in real-world scenarios. Furthermore, the images were standardized by scaling each channel using predetermined mean and standard deviation values to match the data distribution used during the model training process.

**Figure 2:**
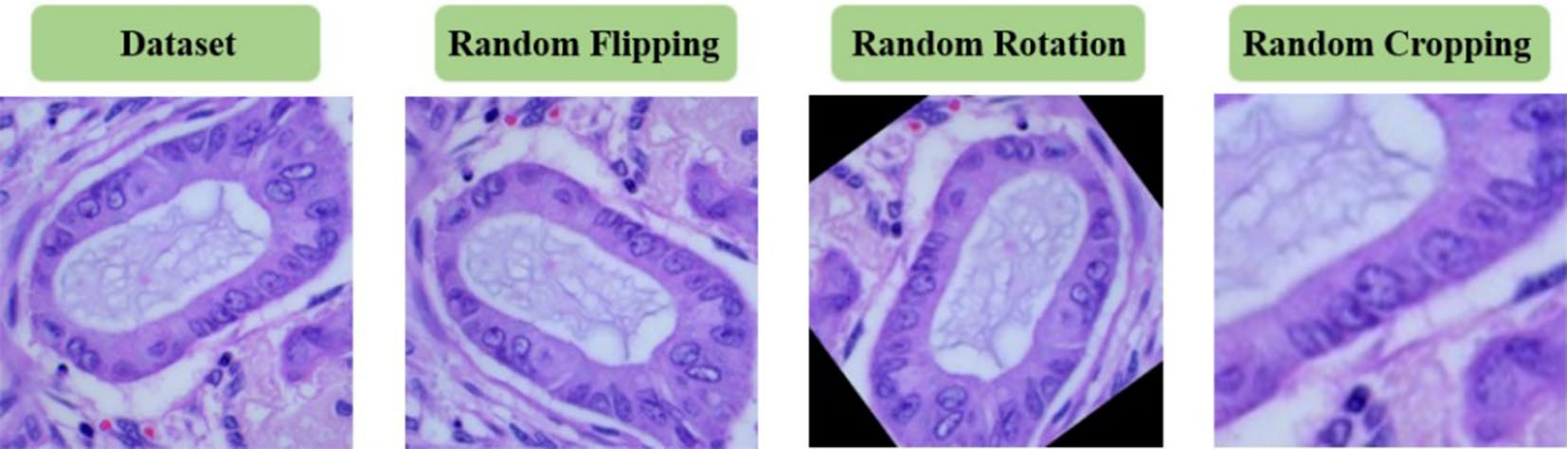
Dataset preprocessing display.

For preprocessing the test dataset, we adopted a more simplified and standardized approach. Specifically, we first resized the images so that their shorter side measured 256 pixels, followed by a center crop to obtain images of 224 × 224 pixels. This ensured consistency in the input size during the model evaluation phase with that of the training phase. The test data were also standardized to maintain the same data distribution as the training data, ensuring fairness and consistency in evaluation. The preprocessed dataset is depicted in Fig. 2.

### Model construction

#### Proposed deep learning model

In recent times, the approach to diagnosing lung cancer through image analysis has received increasing focus. Traditionally, classifying various cancer subtypes has been a time-intensive and laborious process, relying heavily on medical expertise. With the progression of deep learning, automated methods for image analysis have been gradually evolving. Notably, deep neural networks leveraging multiple features, like Convolutional Neural Networks (CNNs)^20^ and traditional Transformer architectures for image classification^21^, have been explored. However, CNN-based models primarily extract local features using smaller convolutional kernels, posing challenges in capturing global contextual features. On the other hand, Transformer-based neural networks, despite their extensive parameter sets, face difficulties in handling sequential data efficiently.

**Figure 3:**
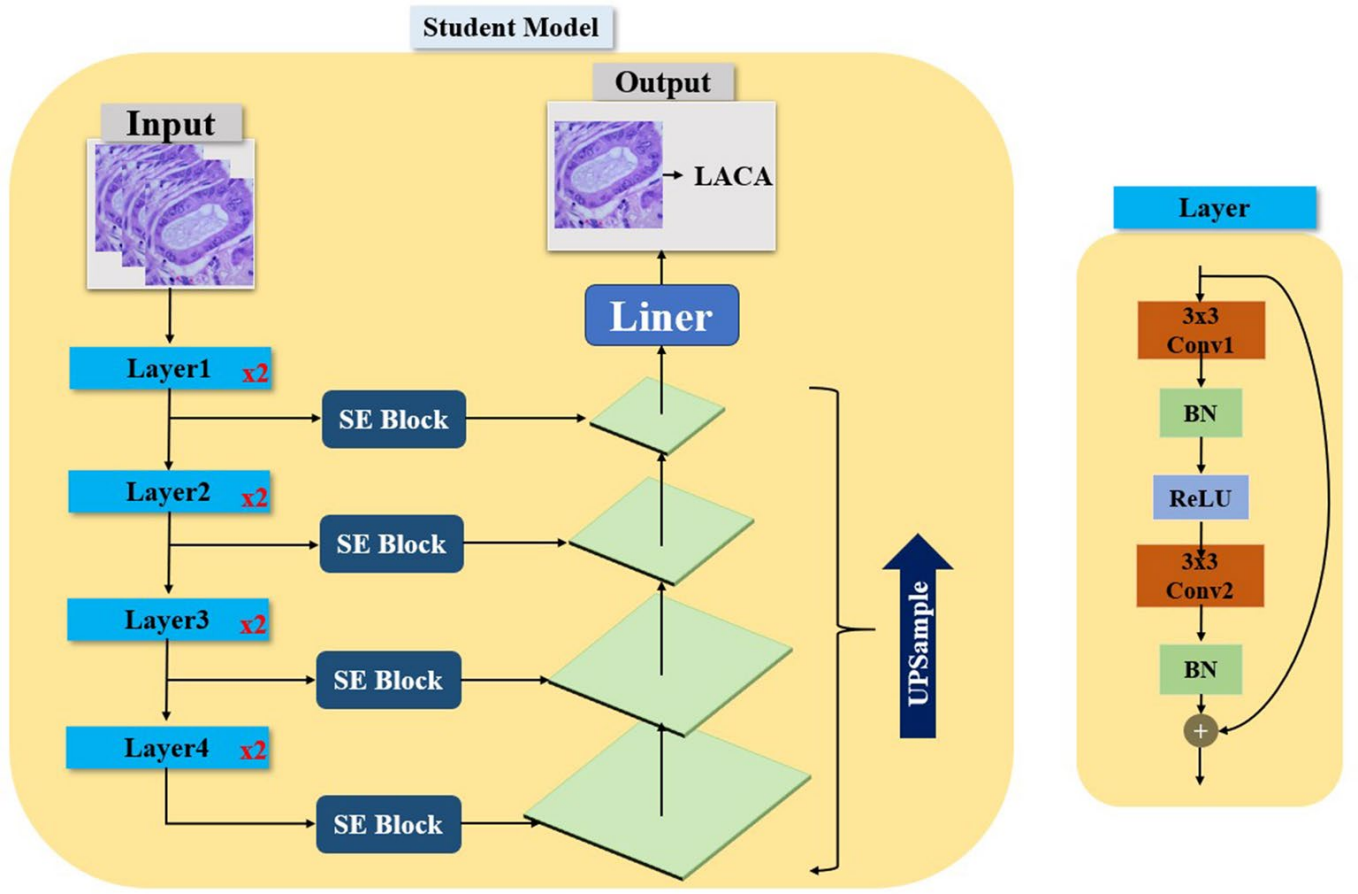
Specific architecture of the model.

To overcome these limitations, we developed the MFStudentLNet model. This model utilizes Resnet18 for feature extraction and integrates FPN at varying scales within each Resnet18 layer. SE modules are inserted between the Resnet and FPN layers to adaptively gauge the significance of each channel in classifying lung cancer. Furthermore, we employ knowledge distillation from the larger teacher model, Resnet50, to the more compact student model. This strategy decreases computational and storage demands while boosting the student model’s accuracy. The smaller student models, with their reduced parameter count and complexity, are more interpretable and understandable, a vital aspect in fields requiring clarity on model decision-making, like medical diagnostics and safety applications. Figure 3 depicts the specific structure of the student model.

This paper introduces a unique network architecture, incorporating residual connections to tackle the issues of vanishing and exploding gradients prevalent in deep networks. By extracting features at multiple levels, the model captures diverse semantic information, with higher-level features identifying broader aspects and lower-level ones detailing finer nuances. Overall, image classification models play a pivotal role in lung cancer classification, potentially improving early diagnosis, treatment outcomes, and patient survival rates, and contributing to enhanced medical workflow, quality, and efficiency in healthcare.

#### Comparing model

This study conducts a comparative analysis of several CNN models, which include MobileNet, a conventional CNN, and ResNet-101. These models are juxtaposed with our hybrid approach, which integrates a ResNet18-based student model and a ResNet50 teacher model. The comparative experiments reveal that the combination of the ResNet18 student model and the ResNet50 teacher model surpasses the performance of ResNet101^22^, indicating the potential advantages of our model configuration.

MobileNet^23^ is distinguished by its lightweight architecture, designed to minimize network parameters. It achieves a balance between accuracy and latency through deep separable convolutions, making it particularly well-suited for environments with constrained computational resources, like mobile devices.

CNNs efficiently capture local features through convolution operations and autonomously learn structural patterns in images by progressively extracting and amalgamating these features. CNNs have shown remarkable performance in various image recognition tasks, including the classification of lung cancer cells.

ResNet-101, a deeper variant in the ResNet series, is known for its introduction of residual blocks. These blocks enable the network to maintain effective training capabilities even as it deepens. Boasting 101 convolutional layers, ResNet-101 is tailored for complex tasks that demand extensive deep feature learning.

### Experiment setup

To ensure the rigor of the experiments and prevent random errors, this study employed five-fold cross-validation in both ablation and comparative experiments. This method mitigates biases resulting from uneven data distribution by averaging results over multiple evaluations. In the ablation experiments, different model optimization strategies were applied to the baseline model to assess the effectiveness of each component. Subsequently, the optimized models were thoroughly analyzed in the comparative experiments, with a particular focus on the performance of different models (such as MobileNet, CNN, ResNet101) in terms of accuracy in lung cancer detection and classification. All model parameters were optimized to ensure stability and prevent overfitting. These experiments were conducted on a Windows operating system using Python 3.8 and Pytorch 1.8.2, supported by an Nvidia RTX3060Ti GPU and an AMD 6900HX CPU. Table 1 shows all the training details, with a description.

**Table 1:** Hyperparameter on experiment.

Hyperparameter	Value	Description
Learning rate	0.001	Starting learning rate for the optimizer
Learning rate decay	Reduce by 20% every 10 epochs	Learning rate decreases by 20% every 10 epochs
Batch size	32	Number of samples per forward/backward pass
Optimizer	Adam	Optimizer for automatic learning rate adjustment
Loss function	Softmax	Loss function used for multi-class classification
Kernel size	3 × 3	Kernel size for the convolutional layers
Stride	1	Stride for the convolutional layers
Scheduler Gamma	0.7	Gamma parameter in the learning rate scheduler for adjusting the learning rate
Normalization (mean)	[0.485, 0.456, 0.406]	Mean values for image normalization
Normalization (standard deviation)	[0.229, 0.224, 0.225]	Standard deviation values for image normalization

### Model evaluation

In this study, to comprehensively evaluate model performance, we meticulously selected several metrics, including Accuracy, Matthews Correlation Coefficient (MCC), Precision, Recall, and F1-score. Accuracy was chosen to reflect the overall performance of the model, representing the proportion of correct predictions made. Precision was selected to gauge the quality of the model's positive class predictions, i.e., the proportion of actual positive samples among those predicted as positive. Recall assesses the extent to which the model correctly identifies positive samples, representing the proportion of correct positive predictions out of all actual positive samples. The F1-score, as the harmonic mean of Precision and Recall, serves to balance these two metrics, making it particularly suitable for scenarios where both Precision and Recall are equally important.

Moreover, we employed the MCC as part of our performance evaluation because it offers a comprehensive metric ranging between – 1 and 1, where 1 indicates perfect prediction, 0 signifies random prediction, and – 1 represents entirely incorrect prediction. MCC takes into account all four elements of the confusion matrix (true positives, false positives, true negatives, and false negatives), rendering it a reliable performance assessment tool, especially in imbalanced datasets. The calculation methods for all metrics are shown in formulas (1)–(5).

1 $$\begin{array}{c}Accuracy=\frac{TP+TN}{TP+TN+FP+FN}\end{array}$$

2 $$\begin{array}{c}MCC=\frac{TP\times TN-FP\times FN}{\sqrt{(TP+FP)(TP+FN)(TN+FP)(TN+FN)}}\end{array}$$

3 $$\begin{array}{c}Precision=\frac{TP}{TP+FP}\end{array}$$

4 $$\begin{array}{c}Recall=\frac{TP}{TP+FN}\end{array}$$

5 $$\begin{array}{c}F1-score=\frac{2\left(precision\times Recall\right)}{precision+Recall}\end{array}$$

The rationale behind selecting these metrics was to reflect the model's performance from various angles comprehensively and reveal the model's strengths and limitations in specific tasks. By elucidating the different metrics, we gained a deeper understanding of the multi-dimensional nature of model performance and its impact on practical applications. This multi-faceted analytical approach has facilitated a comprehensive evaluation and improvement of model performance, enhancing our understanding of the model's behavior and decision-making process.

## Results

### Ablation experiment results

In this study, our ablation and comparative experiments were rigorously conducted using a five-fold cross-validation method. This involved randomly splitting the entire dataset into five equally sized subsets. For each experiment round, one subset was designated as the test set, and the remaining four were combined as the training set. We trained and evaluated the model on each unique pairing of training and test sets. This iterative process, conducted five times with varying test sets, allowed us to obtain a comprehensive set of performance evaluations, from which we calculated the mean and standard deviation. This approach provided a robust assessment of the model’s stability and reliability.

The experimental setup also entailed specific training parameters. The learning rate was initially set at 0.001 and reduced by 20% every 10 epochs. We utilized the Adam optimizer for its efficiency in automatic learning rate adjustment. The softmax function was chosen as the loss function, a suitable choice for multi-class classification challenges. The batch size was set to 32, balancing the training speed against memory usage. Each model was iterated until performance plateaued, ensuring the validity of our results.

In our ablation study, the baseline model was ResNet18. We augmented this model by integrating the FPN for extracting multi-scale features, combining inputs from both shallow and deep layers of ResNet18. SE modules were inserted between the FPN and the original layers of ResNet18 to extract attention mechanisms across different levels. Finally, we applied model distillation, using ResNet50 as the teacher model to enhance the learning capabilities of our proposed student model. These modifications were aimed at improving the model's feature extraction and representational capacity, thereby boosting overall performance. Table 2 details the ablation experiment results with the inclusion of these various modules.

**Table 2:** Results of the ablation experiments.

Step	Architecture	Precision	MCC	Accuracy	Recall	F1
Step 1	Baseline	0.8029	0.7210	0.8013	0.7635	0.7827
Step 2	Step1 + FPN	0.8950	0.8361	0.8880	0.8796	0.8872
Step 3	Step2 + SEBlock	0.9149	0.8613	0.9027	0.8881	0.9013
Step 4	Step3 + model distillation	0.9885	0.9827	0.9884	0.9883	0.9884

The results of the ablation experiments confirmed that the model components proposed in this paper significantly enhance the model's precision, thereby validating their effectiveness. Notably, the introduction of the FPN layers and the application of Model Distillation techniques played a substantial role in improving the overall performance of the model. The FPN layers, with their multi-scale feature extraction capability, effectively enhanced the model's processing power. In addition, model distillation, by transferring complex knowledge to smaller and more efficient models, further boosted the model's performance. These enhancements underscore the importance of adopting advanced architectures and techniques in deep learning models to improve efficiency and accuracy.

**Figure 4:**
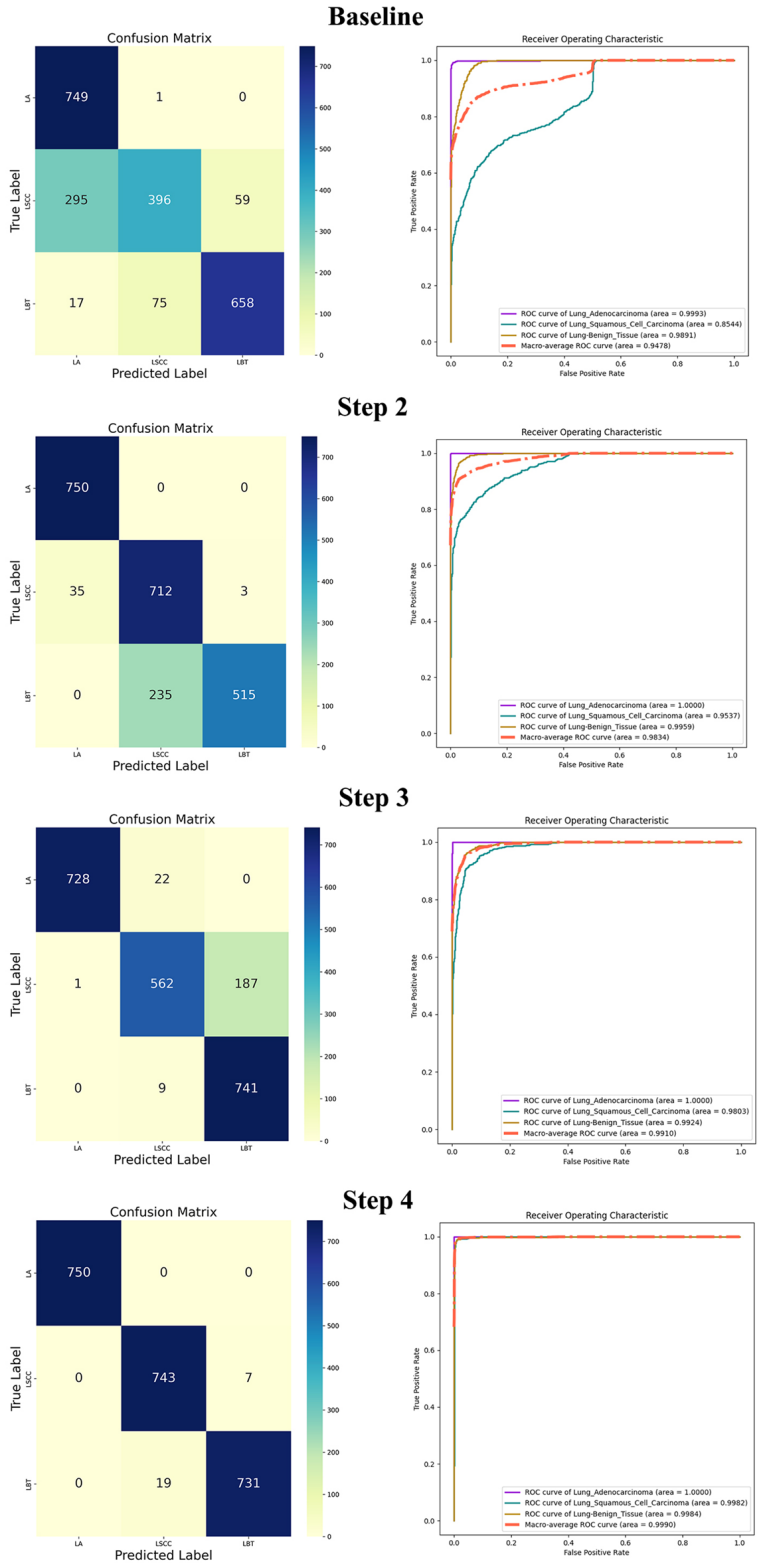
ROC curves and confusion matrices from the ablation experiments.

On the other hand, for a more visual representation of the model's performance, this paper presents the classification confusion matrix results for different ablation components and their corresponding Receiver Operating Characteristic (ROC) curves, as illustrated in Fig. 4.

### Comparative experiment results

In the field of lung cancer diagnostics, our research extended beyond the fundamental implementation of CNN models to a detailed comparison of advanced architectures like MobileNet and ResNet101. The key goal of this comparative analysis was to assess and juxtapose the efficacy of these diverse models in lung cancer detection tasks. Known for its lightweight and efficient design, MobileNet is particularly suited for environments with computational constraints, while ResNet101 is acclaimed for its depth and complexity, excelling in detailed feature capture and learning. This comparative study aimed to highlight the advantages and limitations of each model in processing lung cancer image diagnostics, thus providing an informed basis for selecting the most appropriate model for clinical use. This approach allowed for a more comprehensive understanding of the viability and impact of different network structures in automated lung cancer diagnosis.

In our research, we implemented five-fold cross-validation for experiments and outlined the thorough results in Table 3. This encompassed an in-depth comparative analysis of ResNet101, CNN, MobileNet, VIT, and our proposed model, inclusive of each model's evaluation metrics. To maintain consistency and rigor in the experimental results, the parameters and settings for the comparative experiments were kept uniform with the ablation study. This ensured standardization across all model evaluations, allowing for an equitable comparison of their performances. The application of five-fold cross-validation further bolstered the reliability and stability of our findings, solidifying the scientific validity and accuracy of our conclusions.

**Table 3:** Comparative experiment results table.

	Recall	Precision	Accuracy	MCC	F1	Times (s)
Mobilenet	0.8785	0.7937	0.8529	0.8249	0.8339	93
CNN	0.8918	0.8143	0.8684	0.8446	0.8513	83
Resnet101	0.9809	0.9709	0.9804	0.9799	0.9759	183
Desnet121	0.9632	0.9638	0.9636	0.9455	0.9635	127
Resnet50	0.9448	0.9490	0.9471	0.9217	0.9469	128
VIT	0.9063	0.8545	0.9013	0.8964	0.8796	244
Our	0.9885	0.9827	0.9884	0.9883	0.9856	91

As shown in Table 3, based on the results of the comparative experiments, it's evident that our model demonstrates a significant advantage over the MobileNet, CNN, ResNet101, and VIT models across various evaluation metrics. Specifically, our model exhibits the highest Recall, indicating superior sensitivity in identifying positive classes, such as lung cancer cells. Furthermore, it also achieves the highest Precision among all models, implying greater accuracy and a lower false-positive rate in predicting positive classes. In terms of Accuracy, our model leads as well, indicating the highest proportion of correct classifications across all tasks. Finally, our model scores the highest in the MCC, a balanced performance metric that considers true positives, false positives, true negatives, and false negatives, indicating robust classification performance under various conditions.

Additionally, the data in Table 3 indicates that our model requires only 91 s for training per epoch, which is significantly superior compared to all other models evaluated. Or training duration is notably shorter when compared with other high-accuracy models such as Resnet101 (183 s) and VIT (244 s), while still maintaining an exceptionally high precision rate (0.9827) and accuracy (0.9884). Although the training time for the CNN model exceeds that of our model by 2%, our model achieves an improvement in precision of over 10% relative to the CNN. This performance not only highlights the success of our model in computational optimization but also showcases its exceptional balance of performance and efficiency.

**Figure 5:**
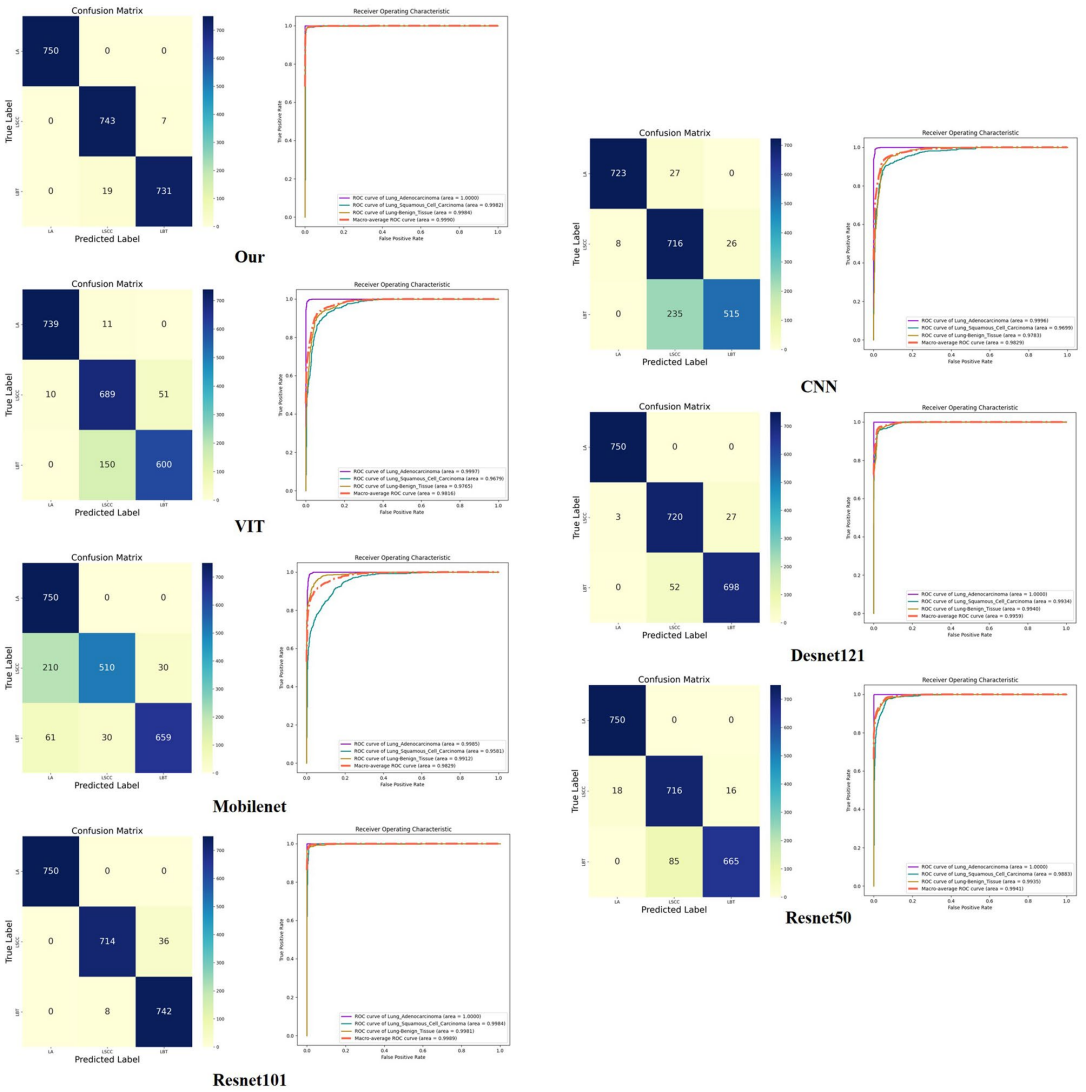
Results of the comparative experiments.

In conclusion, the comparative experiments validate that our model's exceptional performance on these key indicators makes it highly reliable and accurate for lung cancer detection tasks, crucial advantages for selecting a lung cancer diagnostic model. Additionally, we have illustrated the ROC curves and confusion matrix results of the different comparative models in Fig. 5.

### Generalization experiment results

To further verify the model's ability to generalize across different demographic groups and categories, we have expanded the scale of our data. This measure aims to assess the model's generalizability, ensuring that it maintains stable performance in a diversified data environment. In our experiments on generalization capabilities, we not only focus on the task of lung cancer classification but also extend our research to other diseases such as colon adenocarcinoma on the basis of public datasets, thereby enhancing the diversity of the dataset. Our goal is to validate whether the model can exhibit superior performance on a more varied dataset through this approach.

Accordingly, we have supplemented the original dataset with two new categories: colon adenocarcinoma and benign colon tissue, with 5,000 images for each category, thus increasing the total dataset size to 25,000 images. The choice of hyperparameters and the detailed configuration of the model remain consistent with previous comparative trials and ablation studies. On this expanded dataset, we conducted a comparative analysis against different datasets previously used, with the results displayed in Table 4.

**Table 4:** Experiments to evaluate generalization capacity.

	F1	Precision	Accuracy	MCC	Recall
Mobilenet	0.9474	0.9508	0.9475	0.9352	0.9440
CNN	0.9189	0.9258	0.9195	0.9012	0.9121
Resnet101	0.9764	0.9764	0.9765	0.9707	0.9764
VIT	0.8923	0.9013	0.8920	0.8672	0.8835
Resnet50	0.9451	0.9513	0.9453	0.9333	0.9389
Desnet121	0.9660	0.9689	0.9661	0.9585	0.9631
Our	0.9800	0.9812	0.9800	0.9753	0.9788

**Figure 6:**
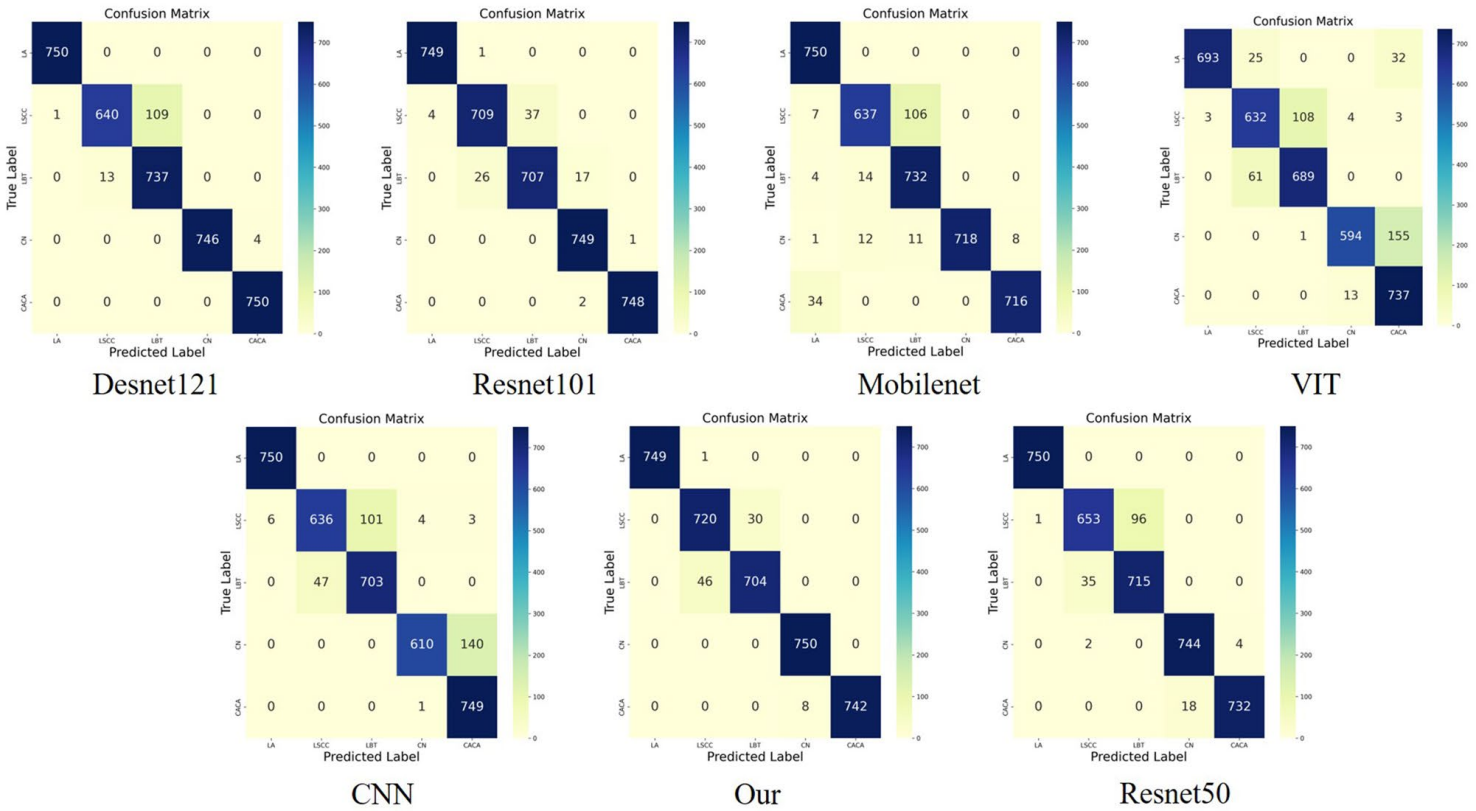
Results of the generalization experiments.

Concurrently, the results depicted in Fig. 6 indicate that the augmentation of the dataset's diversity and size can validate the generalization ability of our model. Specifically, by expanding the dataset, the model is capable of learning a broader range of variations and features, thereby maintaining a high level of recognition capability under various conditions, which is crucial for diverse scenarios encountered in practical applications. Consequently, our model has demonstrated potential for application in a wider and more variable environment.

### Model interpretability

In the diagnosis and treatment of lung cancer, accurately distinguishing between lung adenocarcinoma, squamous cell carcinoma of the lung, and benign lung tissue is crucial. These types of lung lesions differ significantly in their cellular characteristics, pathological features, and progression of the condition. These differences determine the treatment plan and prognosis for patients. Lung adenocarcinoma typically originates from glandular cells in the lungs and is characterized by nuclear atypia and distinct glandular structures. Squamous cell carcinoma of the lung, arising from the squamous cells covering the respiratory tract, is characterized by cell keratinization and prominent intercellular bridges. In contrast to these two malignant pathologies, benign lung tissues exhibit normal cellular arrangement and structure.

**Figure 7:**
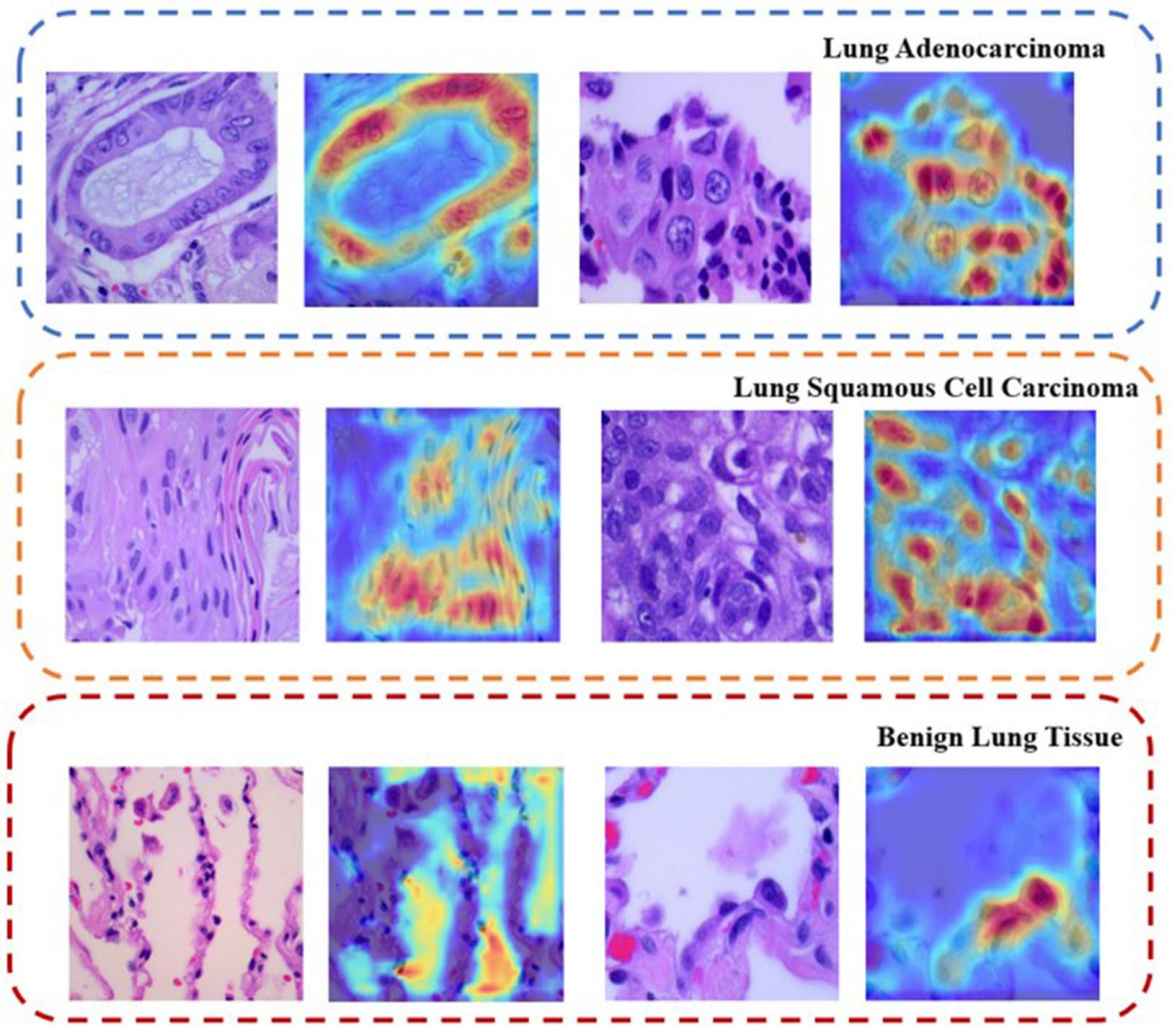
Model's recognition capability for lung cancer pathology.

Given this context, the performance of our model in differentiating these types of lung lesions is particularly critical, directly impacting whether patients receive accurate diagnoses and timely, effective treatment. In Fig. 7, we demonstrate the model's ability to identify lung adenocarcinoma, squamous cell carcinoma, and benign lung tissues. The first row in Fig. 7 represents lung adenocarcinoma, the second squamous cell carcinoma, and the third normal cells. By contrasting the original images with the model’s focus areas at deeper layers, we demonstrate how the model recognizes and differentiates lesions at different levels, revealing the distribution of the model's specific focal points. In these heatmaps, the model's sensitivity to cellular morphology is particularly evident. In the images of lung adenocarcinoma and squamous cell carcinoma, the model focuses on cellular areas with potential pathological significance. The variations in color intensity in the heatmaps highlight these areas, with warmer tones like red and yellow indicating high attention, often around the nuclei, where tumor cell mutations are most apparent. The degree of focus correlates with the potential malignancy of the cells, providing very specific visual clues pointing to potential malignant changes identified by the model.

Conversely, the heatmaps for normal cells show a relatively uniform distribution of blue and green, indicating a more dispersed attention without distinct focal areas. This low-intensity color distribution corresponds with the stable structure of normal cells and their lack of malignant features. Through this contrast, the model not only identifies the differences between pathological and normal cells but also reveals changes within the cells at a finer level.

## Discussion

In this research, we developed a model tailored for differentiating various lung cancer cell types, achieving substantial model optimization through a series of strategies. The incorporation of a multi-channel FPN enhances the model's capability to process complex imagery. Concurrently, the integration of SE (Squeeze-and-Excitation) modules facilitates multi-scale analysis of each channel's significance in classifying lung cancer, bolstering the model's accuracy. Knowledge distillation from larger teacher models, like ResNet50, to more compact student models, ensures a balance between reducing computational and storage demands and maintaining precision. Overall, the proposed model in this study skillfully combines multiple advanced techniques to effectively balance accuracy, computational efficiency, and interpretability, crucial in vital fields such as medical image analysis.

Precise identification of lung cancer cell types offers enhanced diagnostic accuracy for disease confirmation and classification, supporting more informed clinical decision-making by physicians. Such models contribute to the early detection of abnormal changes in lung cancer cells, enabling timely intervention and potentially increasing patient survival and recovery rates. In pathological assessments, manual evaluations can carry inherent subjectivity and errors. Automated classification models reduce such discrepancies, providing more consistent and reliable analysis. The data obtained from accurate classifications are invaluable for lung cancer research, aiding the development of novel medications and treatment approaches.

Compared to prior research, the model introduced in this study shows notable advancements in accuracy. We conducted a comparative analysis with several lung cancer classification models from existing literature, including Support Vector Machine (SVM), Random Forest, K-Nearest Neighbors (KNN), and CNN-RNN networks^24–28^. As indicated in Table 5, which presents a comparison of accuracy results, our model demonstrates exceptional performance relative to earlier experimental outcomes. This not only asserts the pioneering nature of our research but also corroborates its effectiveness.

**Table 5:** Comparison of models from previous studies.

Architecture	Accuracy (%)
CNN-RNN	97
SVM	32
RF	88
KNN	87
Our	98.85

While there have been positive advancements in the performance of the tri-classification model for lung cancer, it's important to note that the model's performance heavily relies on the quality and quantity of the training data. Insufficient or biased training data could impact the model's accuracy and generalization capabilities. Additionally, the model's external testing is currently limited, so enhancing its external generalizability on multiple datasets in future work is of utmost importance. Moreover, the decision-making process of deep learning models can be challenging to interpret, which may pose issues in the medical field. Physicians and patients often require a clear understanding of the basis for diagnoses. Therefore, future work should also focus on improving the interpretability of the model.

## Conclusions

In this study, we developed an automated classification scheme specifically for diagnosing lung cancer cells in medical imaging. By incorporating FPN and SE modules for multi-scale feature extraction and employing knowledge distillation techniques from larger teacher models to more compact student models, we significantly improved the accuracy of lung cancer cell classification. Ablation studies validated the importance and utility of these model components. Comparative analyses with traditional medical cell classification models like MobileNet, CNN, ResNet101, VIT, Densenet121, and ResNet50 demonstrated that our method achieved substantial improvements across multiple performance metrics, with 98.84% of images correctly classified, underscoring the efficiency and feasibility of our system in the cytodiagnosis of lung cancer cells. Moreover, testing on larger datasets revealed the model's excellent generalizability, with an accuracy rate of 98%, further confirming its potential in practical applications. However, the lack of high-quality lung cancer pathology databases limits the assessment of the model's universality. Therefore, future work should focus on enhancing the model's interpretability and generalizability across diverse datasets to support its application in a broader medical context, advancing personalized medicine and precision treatment.

This study, by integrating advanced machine learning techniques with medical imaging, has not only advanced the development of lung cancer diagnosis but also set a precedent for applying similar methods across a broader spectrum of medical fields. The success of this automated classification system in accurately diagnosing lung cancer from medical images highlights the potential of AI tools to revolutionize healthcare, offering faster and more accurate diagnoses and personalized treatment plans. This could lead to earlier detection of lung cancer, thereby increasing treatment success rates and improving patient outcomes.

Furthermore, the methodological framework established in this research can be applied to other types of cancer detection and various medical applications, fostering innovation in medical diagnostics and treatment planning. It paves the way for the development of universal diagnostic models that can be applied across different types of medical data and diseases, contributing to the broader field of precision medicine. By enhancing the model's interpretability and generalizability, we are moving towards a future where AI-driven diagnostics become an integral part of clinical practice, thus improving healthcare services on a global scale.

## Data Availability

The datasets used and analysed during the current study are available from the corresponding author on reasonable request.
